# Is cup positioning easier in DDH patients previously treated with Bernese periacetabular osteotomy?

**DOI:** 10.1186/s13018-020-02001-0

**Published:** 2020-10-29

**Authors:** Yunqing Ma, Dianzhong Luo, Hui Cheng, Kai Xiao, Wei Chai, Rui Li, Hong Zhang

**Affiliations:** 1 Department of Orthopedics, The Fourth Medical Centre of Chinese PLA General Hospital, Beijing, 100048 China; 2 Department of Orthopedics, The First Medical Centre of Chinese PLA General Hospital, Beijing, 100853 China

**Keywords:** Acetabular wall defect, Periacetabular osteotomy, Total hip arthroplasty

## Abstract

**Background:**

Acetabular orientation changes after periacetabular osteotomy (PAO) lead to technical change when performing subsequent total hip arthroplasty (THA). There is no unified consensus regarding the solution for acetabular component installation after PAO. In the current study, we performed computed tomography (CT)-based simulation of acetabular component installation and compared the acetabular defect and component position following THA after PAO and the same patient before PAO.

**Methods:**

From January 2014 to December 2018, pelvic models of 28 patients (28 hips) underwent PAO and with the risk factors to develop secondary osteoarthritis. The acetabular reconstruction process was simulated using 3D models from CT data, and the acetabular component coverage was calculated in 3D space based on the measurement and algorithm we proposed. We evaluated the anterior, posterior, superior, inferior acetabular sector angle (ASA), the medial wall thickness (MWT), and the distance from the hip center to the plane of pubic symphysis and ossa sedentarium in the study group (post-PAO group) and control group (pre-PAO group). In addition, we investigated the changes in the acetabular component covering and size between the two groups.

**Results:**

A-ASA and I-ASA values were significantly smaller in the post-PAO group than in the pre-PAO group. The S-ASA and distance values were significantly bigger in the post-PAO group. Compared to the pre-PAO group, the post-PAO group has a bone defect in the anterior and inferior medial. However, the post-PAO group has to elevate the cup to improved component coverings.

**Conclusion:**

Acetabular defection following simulation of cup installation after PAO was significantly changed compared to those without PAO. Elevation of hip joint centers as much as 4 mm and increase acetabular cup anteversion were therapeutic options for DDH patients following THA after PAO

## Introduction

Periacebular osteotomy (PAO) is considered suitable to treat acetabular dysplasia in young adults with developmental dysplasia of the hip (DDH) in order to prevent the progression of osteoarthritis. PAO was first described by the Ganz in the mid-1980s; it is also known as the Bernese periacetabular osteotomy. Long-term follow-up after surgery revealed that patients would still develop secondary osteoarthritis, and nearly 2/3 of patients needed total hip arthroplasty (THA) [1, 2]. There are many risk factors leading to the failure of PAO. Wells et al. considered that patients older than 25 years, obvious preoperative osteoarthritis symptoms, and the joint space less than 2 mm or more than 5 mm were the main risk factors for postoperative failure [3]. Charlotte et al. suggested that advanced age, hip instability, postoperative joint space ≤ 3 mm, and lateral center-edge (LCE) angle < 30° and > 40° may lead to the failure of PAO [4]. A few reports describe the altered morphology of the acetabulum in hip dysplasia may complicate the insertion of the THA—even after PAO [5, 6]. The aim of this study was to perform CT-based simulation of acetabular component installation and compared the acetabular defect and component position following THA after PAO and the same patient before PAO, to explore whether PAO would increase the difficulty of acetabular component placement.

## Materials and methods

### Patients and procedures

Patients eligible for inclusion were those who underwent PAO from January 2014 to December 2018 with a minimum 1 year follow-up after PAO. Inclusion criteria were (1) patients age at PAO surgery≥ 30 years, (2) preoperative diagnosis was acetabular dysplasia and only acetabular side received PAO treatment, and (3) LCE angle ≤ 0° before PAO, and with hip instability. Exclusion criteria were (1) preoperative diagnosis was epiphyseal slippage, Legg-Calve-Perthes disease, etc; (2) with previous history of hip surgery; (3) history of hip surgery post-PAO except for internal fixation removal; and (4) patients who had incomplete follow-up radiographic or poor radiographic material. A total of 28 patients (25 females) were eventually included in the study with a mean age of 36.3 ± 5.0 years (range 30.2–47.8 years). The lateral center-edge angle before PAO was − 2.96° ± 3.53° (range − 10°to 0°) and after PAO 20.20° ± 3.53 (range 15° to 37°). There are 14 hips for TÖnnis grade 0, 12 for TÖnnis grade 1, and 2 for grade 2. There are 20 hips for Crowe type I and 8 for type II 8.

### Simulating implantation of the prosthetic acetabular component

**Fig. 1:**
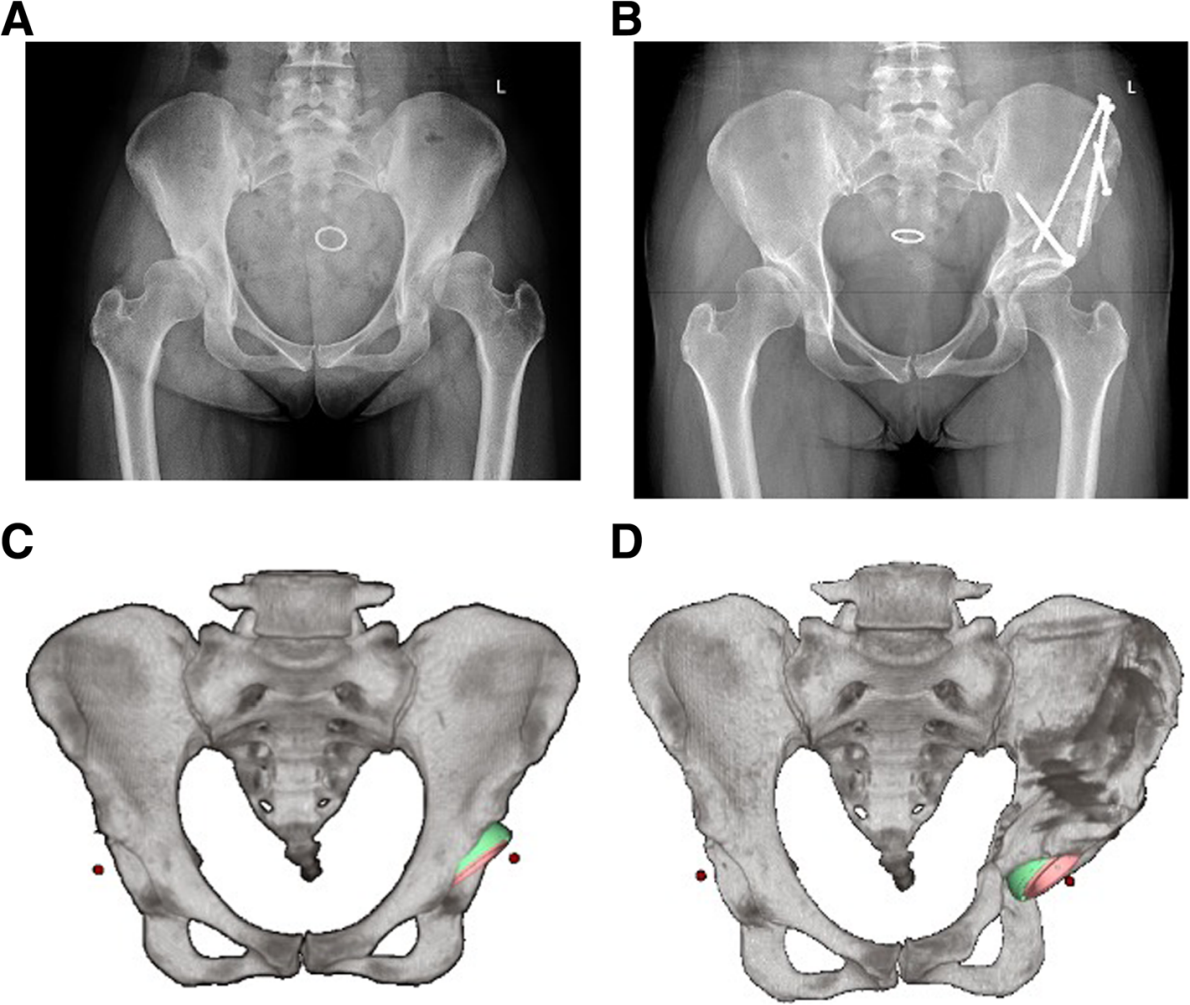
Simulating implantation of the prosthetic acetabular component and the position of the non-cover area during the simulated acetabular prosthesis implantation before and after PAO surgery

A set of hemispherical virtual acetabular components was created according to the sizes of dysplastic acetabula using AI-HIP software (Changmugu, Beijing). The outer diameters of the acetabular components ranged from 38 to 60 mm in 2-mm intervals, and all had a shell thickness of 4 mm. These 3D models were imported into Mimics software. The simulated acetabular replacement was performed by placing the component in the true acetabulum (Fig. 1). The AI-HIP software simulates the optimal position of the acetabular component, and the pelvic position was calibrated by the pelvic anterior plane for angles measurement. AI-HIP software was based on the patient’s acetabular condition, by calculating the acetabular prosthesis coverage and referring to the anatomical rotation center of the hip joint to determine the acetabular prosthesis position. The position of the cup was confined by the peripheral border of the true acetabulum such that the so-called rimfit was achieved. The cup size was chosen to best accommodate the anteroposterior diameter of the true acetabulum, which tended to utilize the osseous peak of the anterior and posterior bone columns in the axial section. The inner cortex of the medial acetabular wall was set as the medial limit for cup placement. After that, the surgeon can finally adjust the prosthesis position. This study fixed acetabular prosthesis with an anteversion of 15° and inclination of 40° [7]. Secondly, refer to the anterior and posterior diameter of the acetabulum to adjust the size of the acetabular prosthesis. In doing this, we strove to maximize the preservation of the native bone of the true acetabulum for morphological evaluation and simultaneously attain optimal osseous coverage (> 75%).

### Acetabular morphologic evaluation of CT simulation

In this protocol, the rotation center of the true acetabulum was determined by the central point of the component. The surface contact area between the cup and native bone was defined as the effective bone mass of the true acetabulum. In this context, effective bone mass corresponds to the bone that could be effectively utilized to support the implanted acetabular component.

**Fig. 2:**
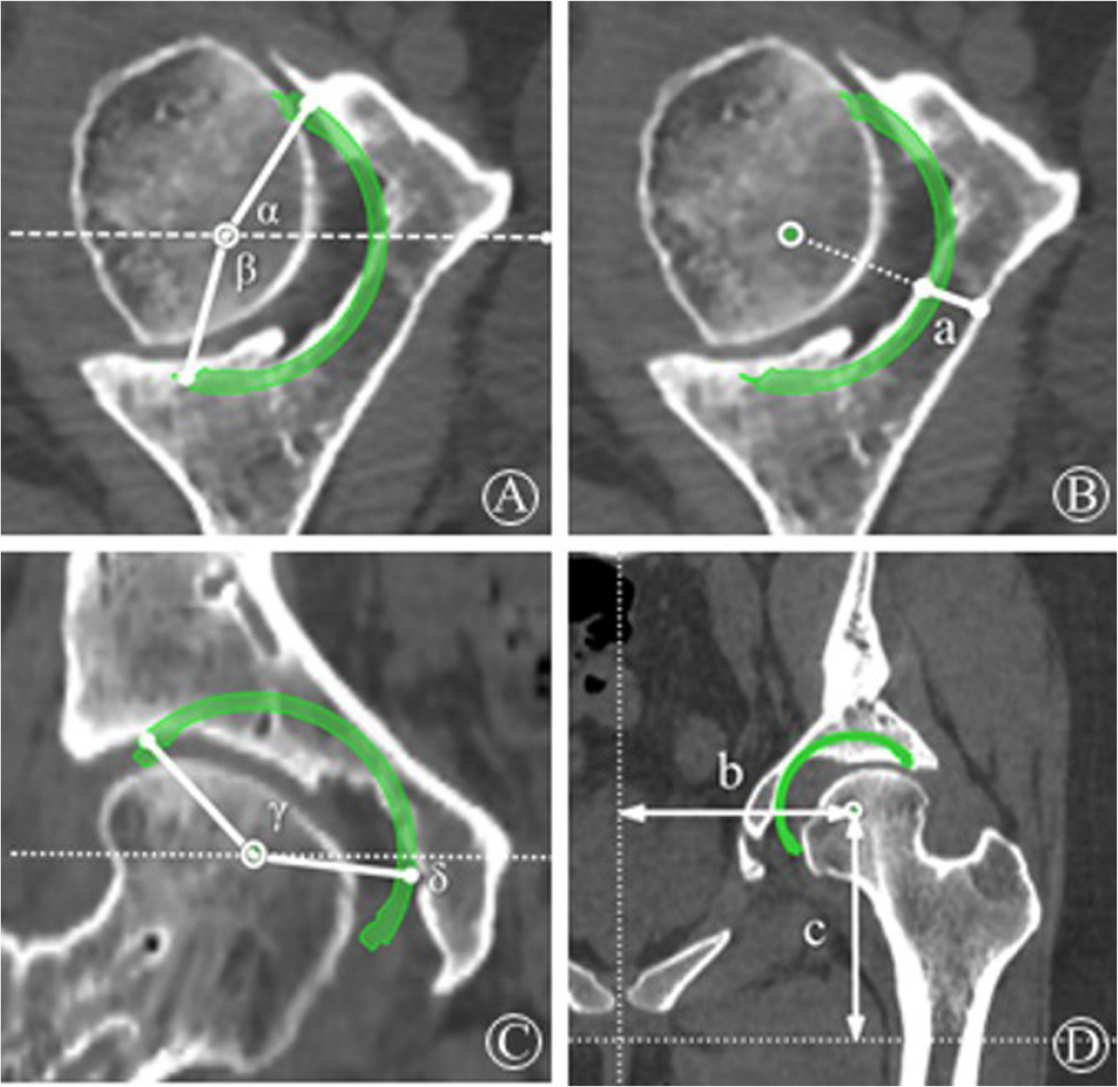
Measurement of bone defect after simulating implantation acetabular prosthesis based on CT scan. **a** A-ASA = α and P-ASA = β are measured on the axial plane through rotation center. **b** The minimum thickness of acetabular inner wall is measured on the straight line passing through the rotation center. **c** S-ASA = γ and I-ASA = δ are measured through the coronal plane of rotation center. **d** On the same plane, the distance from the rotation center to the pubic symphysis (**b**) and the vertical distance (**c**) from the same point the horizontal plane of the lowest point of the ischial tubercle were measured

The acetabular defect was evaluated using the measurement method of Yang et al. [8]. We evaluated the anterior acetabular sector angles (A-ASA) and posterior acetabular sector angles (P-ASA), which are the angles between the most marginal point of contact anteroposterior wall and the parallel line connecting the anterior superior iliac spine, with a central focus on the hip joint center (Fig. 2a). Second, we measured the medial wall thickness (MWT) which is the medial wall length with the axial plane passing through the central focus on the hip joint center (Fig. 2b). Third, to treat the superior and inferior acetabular defect, using the coronal view and defined the superior ASA and inferior ASA, which are the angles between the original superior and inner inferior wall with a central focus on the hip joint center (Fig. 2d). Additionally, we evaluated acetabular defects with simulating component implantation and compare the ratio of the contact area of the two groups.

### Statistical analysis

To assess interobserver reliability, 2 experienced surgeons independently performed implantation simulation and corresponding measurements. To assess intraobserver reliability, both the implantation and the measurements were repeated by the same surgeon after 1 month. The intraclass correlation coefficient (ICC) was used to calculate interobserver and intraobserver effects.

Kolmogorov-Smirnov tests were performed to determine the distributions of the data. For parametric data, when the variances in the groups were the same, 1-way ANOVA (analysis of variance) was used to compare the differences among groups, followed by the LSD (least significant difference) method for pairwise comparisons. For nonparametric data, or when the variances in the groups were different, Kruskal-Wallis ANOVA was performed, followed by the Dunn test for pairwise comparisons. All statistical analyses were performed using SPSS version 19.0 software (IBM), and a *p* value of < 0.05 was considered to be significant.

## Results

The intraobserver reliabilities, evaluated with 1-way random effects model ICCs, were in the excellent range of 0.80 to 0.92. The interobserver ICCs ranged from 0.85 to 0.92 for the morphological indices. The A-ASA were significantly smaller in the post-PAO group than in the pre-PAO group (54.47° ± 13.08° vs 71.72° ± 4.22°, *p* = 0.000) through the rotating center on the axial plate after simulated acetabular prosthesis implantation. There was no statistical difference between the two groups of the P-ASA (*p* = 0.054). There was no statistical difference of the MWT between the two groups (8.39 ± 2.61 mm vs 7.39 ± 2.84 mm, *p* = 0.220). The S-ASA of the post-PAO group was significantly larger than that of the preoperative group on the coronal plate (139.28° ± 1.95° vs 113.17° ± 8.89°, *p* = 0.000). In addition, the I-ASA of post-PAO group was significantly smaller than the pre-PAO group (8.24° ± 6.46° vs 18.75° ± 3.34°, *p* = 0.000). The mean height of rotation center in the post-PAO group was 28.52 ± 4.70 mm, and the pre-PAO group was 24.04 ± 3.48 mm (*p* = 0.001). The distance from rotation center to the pubic symphysis was 79.92 ± 6.28 mm in the postoperative PAO group, and 82.92 ± 4.67 mm before PAO, there was no statistical difference between the two groups (*p* = 0.062). The component coverage ratio of the hips in the post PAO groups was 84.39 ± 5.13% and 84.95 ± 6.13% in the before PAO group. The cup size of the simulated implantable prosthesis after PAO was greater than that before PAO, and there was a statistical difference between the two groups (*p* = 0.001) (Table 1). Bone defects were located in the anterior and inferior of the post-PAO group, while there is a significant segmental deficiency in the superior and posterosuperior directions in pre-PAO group.

**Table 1:** Comparison of bone defect and position of acetabular prosthesis before and after periacetabular osteotomy

	A-ASA (°)	P-ASA (°)	S-ASA (°)	I-ASA (°)	MWT, mm	VDRC, mm	HDRC, mm	Cover rate, %	Size, mm
**Pre-PAO**	71.72 ± 4.22	100.85 ± 3.56	113.17 ± 8.89	18.75 ± 3.34	8.39 ± 2.60	24.04 ± 3.48	82.91 ± 4.78	84.39 ± 6.13	48
**Post-PAO**	54.47 ± 13.08	102.61 ± 2.35	139.28 ± 1.95	8.24 ± 6.46	7.39 ± 2.84	28.52 ± 4.70	79.74 ± 6.35	84.95 ± 5.13	50
***p*** **value**	0.000	0.054	0.000	0.000	0.220	0.001	0.062	0.736	0.001

*VDRC* Vertical distance of rotation center, *HDRC* Horizontal distance of rotation center

## Discussion

Relevant studies have shown that the acetabular defects resulted from rotation of acetabular bone fragment after periacetabular osteotomy (PAO) may affect the angle for placement of acetabular prosthesis later [9, 10]. In early PAO cases, iatrogenic acetabular impingement was caused by insufficient anterior inclination of the bone fragment, for which revision of total hip arthroplasty (THA) was needed to be performed in a short term. Pavizi et al. [11] followed up 41 patients who had received THA after early PAO. The mean interval between PAO and THA was 6.3 years, and 91% of the acetabular prostheses had 45° of extension and 15° of anteversion. During the operation, some acetabulum was found retroverted, and the surgeon should be reminded of paying attention to the angle for placement of the prosthesis. Current requirements for localization of bone fragment during PAO are as follows: (1) the outer coverage for femoral head shall be restored, (2) the center of rotation for femoral head shall shift inward, and (3) there shall be appropriate anterior coverage of the femoral head. Therefore, recent studies have shown that the anteversion angle of acetabular prosthesis in patients who received THA after PAO failed was greater than in patients of the same type with osteoarthritis secondary to progressive hip dysplasia (DDH) who did not receive PAO [12, 13]. This study showed the same results. As simulating acetabulum prosthesis at fixed 45° of extension and 15° of anteversion, the anterior coverage and inner coverage of acetabular prosthesis was reduced in the post-PAO group, i.e., the A-ASA in imaging measurement of the group was significant smaller than that of the pre-PAO group, but there was no statistical difference in P-ASA between the two groups, which indicated that the anterior coverage of the prosthesis could be increased through increasing the anteversion of the prosthesis without compromising posterior coverage of the prosthesis.

The ideal position for acetabular prosthesis should be the anatomical center of the hip joint. As Crowe I and II patients with advanced osteoarthritis secondary to progressive hip dysplasia (DDH) received THA, the center of rotation would be appropriately shifted upward or inward, so that the acetabular prosthesis coverage could be more than 70% [14, 15]. After Bernese periacetabular osteotomy, the center of rotation for the hip joint was shifted inward. Considering the improvement in the outer coverage for femoral head, theoretically, it was unnecessary to move acetabular prosthesis upwards for increasing the coverage of the prosthesis for later THA. However, previous studies showed that [16] as a type of periacetabular osteotomy, THA treatment after failure of Rotational acetabular osteotomy (RAO) made the rotation center for acetabular prosthesis of the patients about 10 mm higher than the normal anatomical center. Other reports [5, 6, 17] also indicated that the position of acetabular prosthesis after RAO had an upward and outward trend as compared to the normal anatomical position. In this study, a similar situation occurred after RAO. The rotation center of the simulated acetabular prosthesis after PAO was increased by about 4 mm as compared with that before PAO, and the difference was statistically significant. This indicated that as fixing the anteversion angle of the prosthesis, it was necessary to move up the rotation center to increase the contact area between the anterior coverage and the inner wall of the prosthesis. In addition, inappropriate position of bone fragment in PAO, late collapse of bone fragment, and loss of rotation angle would lead to the upshift of rotation center during late THA.

According to the study, to achieve long-term stability, the central-edge angle of acetabular prosthesis after THA should be > 0°, that is, S-SAS in this study should be greater than 90° [18]. In this study, the S-ASA in the pre-PAO group was up to 113° through slight upward movement (< 10 mm) of the prosthesis as simulating acetabular prosthesis implantation, but still significantly lower than 139°, the S-ASA in the post-PAO group. Therefore, the shift of the rotation center upward after PAO larger than that before PAO was not to increase S-ASA, but to improve bone defects in the medial wall and antero-inferior side of the acetabulum. The inner wall thickness of acetabulum at the height from the teardrop to the rotation center of femoral head after PAO were significantly less than that before PAO. Some cases even had complete bone defects at the lower part of the inner wall while many patients with osteoarthritis secondary to DDH got secondary thickened inner wall of the acetabulum as receiving THA due to developmentally thickened medial wall or coverage of the oval fossa by osteophytes. As receiving THA, the rotation center can be shifted inward to meet the angle requirement of S-ASA, i.e., greater than 90°. Although there was no statistical difference in this study, the center of rotation for acetabular prosthesis after PAO showed a tendency to shift inward as compared with that before PAO because the distance for inward shift of PAO bone fragment was much greater than the distance for inward shift of rotation center in DDH patients receiving THA, which resulted in a relative inward shift of the rotation center after PAO.

As simulating prosthesis implantation before and after PAO, if there was no significant difference in acetabular prosthesis coverage, a larger acetabular prosthesis was allowed to be implanted after PAO. A larger diameter of prosthesis could match a larger diameter of femoral ball head or a thicker lining, so as to increase the stability and activity range of joint [19], and so improve the survival rate of prosthesis and postoperative functions for the patients.

This study still has some limitations as follows: (1) The study was about a prosthesis implantation simulated by software based on CT, and the patients’ hip joints were not like the actual circumstances before THA. However, the degree of bone defect was not more severe than that at an early stage for Crowe and II DDH patients with advanced osteoarthritis secondary to DDH. (2) The sample size was relatively small (*n* = 28) for the number of patients with LCE ≤ 0° over 30 years old was limited, and the hip joint CT was not a routine examination for postoperative follow-up. (3) In this study, the front plane of the pelvis was used as the reference for the angle measurement while functional pelvic positions, e.g., inclination of pelvis, were not considered.

In conclusion, A-ASA of acetabular prosthesis for THA after PAO was significantly lower than that before PAO. Although the S-ASA was improved significantly, the anterior coverage and medial coverage of acetabular prostheses were reduced, for which stress analysis and long-term follow-up should be further performed for evaluating relevant influence on long-term stability of prosthesis. The increase in size of prosthesis implantable after PAO may improve the stability and activity of joint. As performing revision of THA after PAO, the coverage rate of acetabular prosthesis can be improved through increasing the anteversion of the prosthesis and moving up the center of rotation for the hip joint.

## Data Availability

The datasets used and/or analyzed during the current study are available from the corresponding author on reasonable request
